# Quantitative proteomics identifies 38 proteins that are differentially expressed in cucumber in response to cucumber green mottle mosaic virus infection

**DOI:** 10.1186/s12985-015-0442-x

**Published:** 2015-12-15

**Authors:** Hua-Wei Liu, Chao-Qiong Liang, Peng-Fei Liu, Lai-Xin. Luo, Jian-Qiang Li

**Affiliations:** Department of Plant Pathology, China Agricultural University, Beijing, 100193 PR China; Beijing Engineering Research Centre of Seed and Plant Health (BERC-SPH), Beijing Key Laboratory of Seed Disease Testing and Control (BKL-SDTC), Beijing, 100193 PR China; Molecular Plant Pathology Laboratory, USDA-ARS, Beltsville, MD 20705 USA

**Keywords:** Cucumber green mottle mosaic virus (CGMMV), *Cucumis sativus* L, iTRAQ, Proteomics, qPCR

## Abstract

**Background:**

Since it was first reported in 1935, *Cucumber green mottle mosaic virus* (CGMMV) has become a serious pathogen in a range of cucurbit crops. The virus is generally transmitted by propagation materials, and to date no effective chemical or cultural methods of control have been developed to combat its spread. The current study presents a preliminary analysis of the pathogenic mechanisms from the perspective of protein expression levels in an infected cucumber host, with the objective of elucidating the infection process and potential strategies to reduce both the economic and yield losses associated with CGMMV.

**Methods:**

Isobaric tags for relative and absolute quantitation (iTRAQ) technology coupled with liquid chromatography-tandem mass spectrometric (LC-MS/MS) were used to identify the differentially expressed proteins in cucumber plants infected with CGMMV compared with mock-inoculated plants. The functions of the proteins were deduced by functional annotation and their involvement in metabolic processes explored by KEGG pathway analysis to identify their interactions during CGMMV infection, while their in vivo expression was further verified by qPCR.

**Results:**

Infection by CGMMV altered both the expression level and absolute quantity of 38 proteins (fold change >0.6) in cucumber hosts. Of these, 23 were found to be up-regulated, while 15 were down-regulated. Gene ontology (GO) analysis revealed that 22 of the proteins had a combined function and were associated with molecular function (MF), biological process (BP) and cellular component (CC). Several other proteins had a dual function with 1, 7, and 2 proteins being associated with BP/CC, BP/MF, CC/MF, respectively. The remaining 3 proteins were only involved in MF. In addition, Kyoto Encyclopedia of Genes and Genomes (KEGG) analysis identified 18 proteins that were involved in 13 separate metabolic pathways. These pathways were subsequently merged to generate three network diagrams illustrating the interactions between the different pathways, while qPCR was used to track the changes in expression levels of the proteins identified at 3 time points during CGMMV infection. Taken together these results greatly expand our understanding of the relationships between CGMMV and cucumber hosts.

**Conclusions:**

The results of the study indicate that CGMMV infection significantly changes the physiology of cucumbers, affecting the expression levels of individual proteins as well as entire metabolic pathways. The bioinformatic analysis also identified several pathogenesis-related (PR) proteins that could be useful in the development of disease-resistant plants.

## Background

*Cucumber green mottle mosaic virus* (CGMMV), which belongs to the *Tobamovirus* genus of the *Virgaviridae* family, is a damaging pathogen of cucurbit crops. The virus is transmitted mechanically, via seeds, pollen and other propagation materials [1], and produces typical mosaic patterning on the leaves of infected plants, as well as fruit distortions [2] that result in a reduced yield and lower market value [3]. Since CGMMV was first reported in the UK in 1935 [4], it has been detected in many other countries including Israel, China, Greece, America and Canada [5–9]. Although the spread of this disease between different countries and regions has been rapid, to date there are no effective control strategies to reduce the economic losses it caused.

The susceptibility of particular plants to disease infection and the severity of the ensuing disease are closely associated with the defense response of the host plants [10]. The invasion of plant pathogens activates various defense mechanisms intended to prevent colonization. These internal changes, often mediated at the transcriptional level, alter gene expression and can also indirectly affect plant performance [11, 12]. Understanding how viruses invade plants and the post-transcriptional mechanisms mediating the interactions between them is fundamental to developing effective management strategies for the control of viral disease in the field. Previous research has shown that both proteins and metabolites of the host plant are involved in the replication of viruses and the successful infection of plant cells [13]. Proteomics is a useful technique for the study of these kinds of interactions and has been widely applied in the identification of proteins induced by abiotic and biotic stress and the characterization of their function during virus infections. For example, 2-DE and LC-MS/MS analysis identified many proteins produced when rice plants were infected with rice yellow mottle virus (RYMV), including those associated with stress related responses, as well as cellular metabolism and host mRNA translation [14]. A similar study identified 203 proteins produced by sugar beet infected with beet necrotic yellow vein virus (BNYVV) [15], most of which were associated with photosynthesis and energy metabolism, in addition to host responses to stimulus and metabolism, while a proteomics approach has also been used to demonstrate the importance of the N resistance gene in the response of *Nicotiana benthamiana* to TMV infection [16]. Many so-called effectors are generated during the interaction of pathogens and their hosts, while the proteins produced by phytopathogens themselves can enter plant cells and trigger host defense mechanisms. For example, it has been found that effector-triggered immunity (ETI) can subvert pathogen-associated molecular patterns (PAMPS) by the recognition of microbial proteins, which can result in the suppression of microbial growth via the activation of protein-mediated resistance [17]. For example, silencing of the host ATP-synthase γ-subunit (AtpC) and Rubisco activase (RCA) genes in *N. benthamiana* resulted in the accumulation and pathogenicity of TMV in its leaves [18], while the overexpression of the hexose transporter gene *LeHT1* in R tomato plants restricted the development of the tomato yellow leaf curl virus (TYLCV) [19]. Research has shown that there is a great diversity of such effectors, which can be either intracellular or extracellular components, and that differential expression of the proteome and post-transcriptional regulation can play an important role during infection of host cells by viruses. Consequently, proteomics approaches to investigate host pathogen interactions have attracted increasing attention in recent years [20].

Proteomic analysis can generate an abundance of information on the individual proteins involved in biological responses, while the combination of differential-expression proteomics with novel techniques using isobaric tags for relative and absolute quantitation (iTRAQ) provide an opportunity to investigate proteins at the transcriptional level. High-throughput techniques such as iTRAQ are particularly valuable as they can be used to evaluate gene expression across the entire genome. In contrast to 2D electrophoresis, the iTRAQ method can be used to directly identify and compare the relative quantity of proteins derived from different tissues in a single experiment. The technique utilizes isobaric tags linked to different reporter ions, which attach to the side chains of lysine residues and the N-terminus of peptides resulting from a protein digest. Mass spectrometry can then be used to determine the ratio of labeled peptides from different sources and thus provide quantitative data [21]. Furthermore, iTRAQ has the additional advantage of resolving very large (>200 kD) and very small molecules (<10 kD), which can be lost during electrophoresis, as well as reducing the degree of analytical bias as a result of the large number of isoelectric points [22]. The application of iTRAQ therefore more closely reflects the actual situation during pathogen infection. The iTRAQ technique has been used to examine the differential expression of proteins in many studies, and the combination of iTRAQ and liquid chromatography-mass spectrometry/mass spectrometry (LC-MS/MS) can reveal detailed information regarding virulence factors and pathogenicity [23]. Indeed, a recent study of Sugarcane mosaic virus (SCMV), identified 65 and 59 phosphoproteins in resistant and susceptible genotypes of maize (*Zea mays* L), respectively. Further analysis revealed that many of the phosphoproteins were differentially expressed during SCMV infection, which altered major cellular processes as a consequence of the phosphorylation status of the proteins. Such studies are extremely useful to expand our understanding of the mechanisms of plant-virus interactions [24]. Another study of the Plum pox virus (PPV) in NahG-expressing *N. benthamiana* plants identified more than 1000 non-redundant proteins, 23 of which were differentially expressed in response to viruses expressing a modified P1 serine protease relative to the wild-type virus [25]. The current study utilized a similar approach using iTRAQ coupled with LC-MS/MS to compare the proteome of cucumber plants infected with CGMMV with healthy mock-inoculated control plants.

## Results

### Confirmation of CGMMV infection

**Fig. 1:**
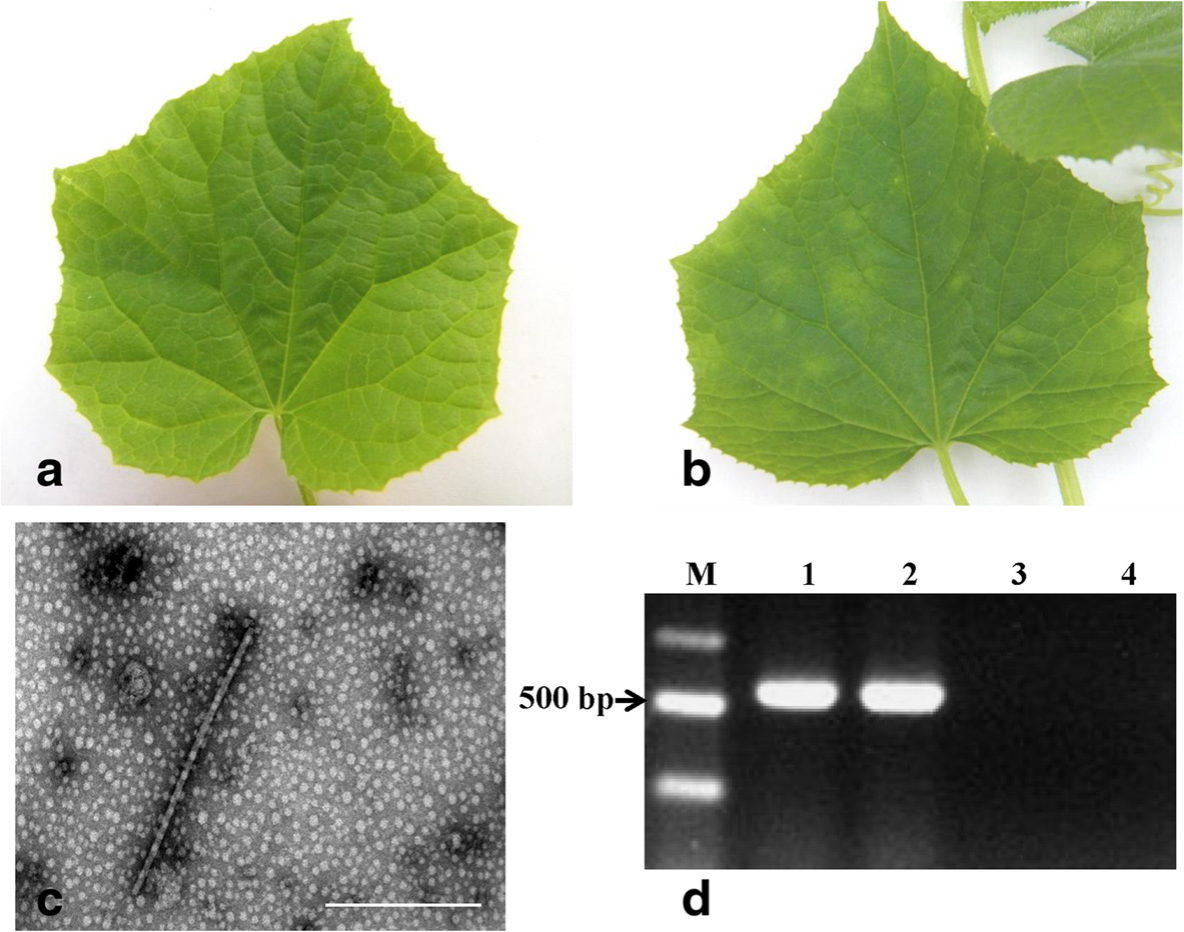
Confirmation of CGMMV status of cucumber seedlings (cv. Zhongnong 16). **a**-**b** Representative leaves used for the iTRAQ analysis. **a** Healthy leaf from mock-inoculated plant. **b** Leaf from CGMMV-infected plant exhibiting typical symptoms of chlorosis and mottling. **c** CGMMV particles from infected leaves observed by SEM, (Bar = 200 nm). **d** Confirmation of CGMMV status by RT-PCR: Lane M: DNA ladder; Lane 1: Positive control (infected leaf obtained from CAIQ); Lane 2: Inoculated sample; Lane 3: Mock-inoculated sample; Lane 4: Negative control (ddH_2_O)

At 45 days post inoculation (dpi), typical symptoms of CGMMV were observed in the leaves of the inoculated seedlings, which exhibited chlorosis and mottling (Fig. 1b). The mock-inoculated leaves (using phosphate buffer as inoculum) remained healthy and exhibited no symptoms of disease (Fig. 1a). The presence of CGMMV particles in the inoculated leaves was confirmed by SEM (Fig. 1c), while the CGMMV status of both the inoculated and mock-inoculated samples was confirmed by RT-PCR, in which only the inoculated samples produced PCR products (Fig. 1d).

### Protein identification and quantification

**Fig. 2:**
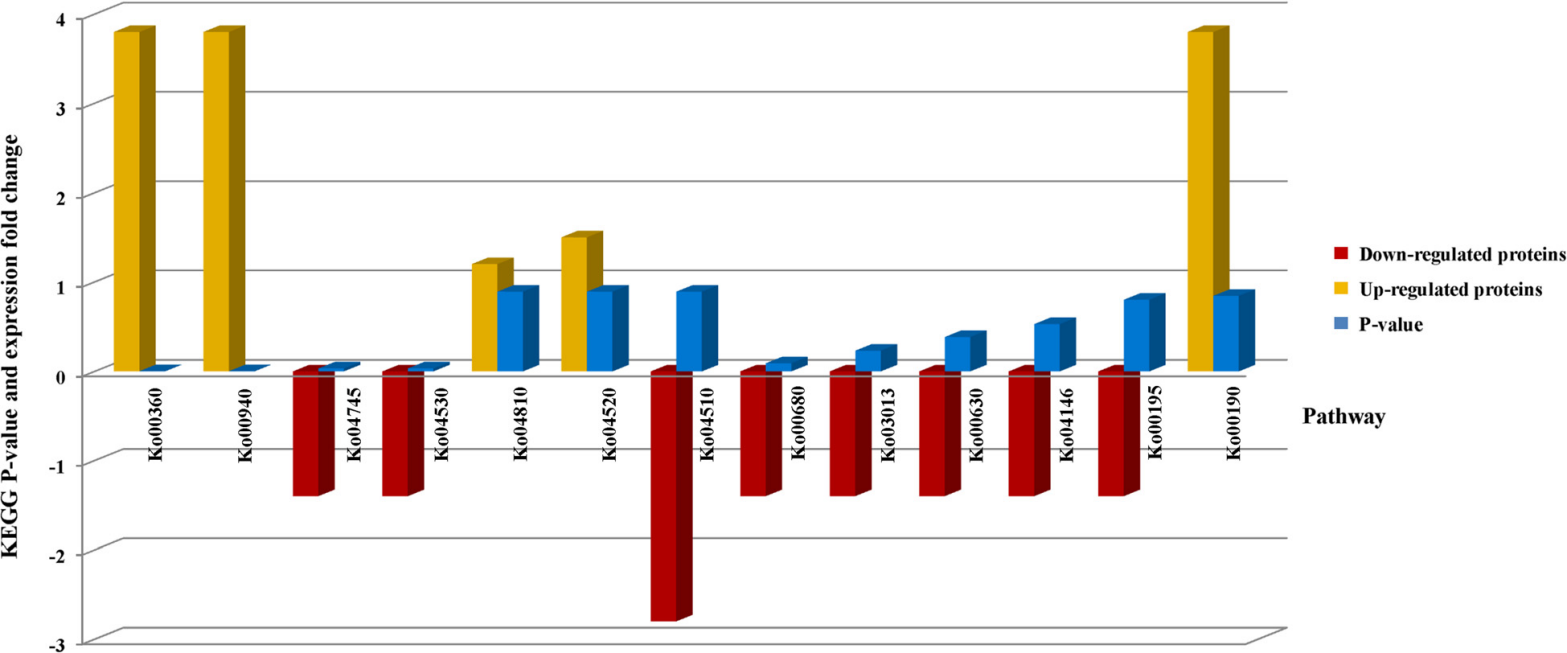
Results of KEGG analysis, which revealed 13 different pathways that are affected by the altered expression of 18 proteins in cucumber plants infected with CGMMV. The orange columns indicate KEGG pathways that involve the up-regulation of proteins while the red columns represent those that involve down-regulation. The blue columns represent the degree of differential expression in CGMMV-infected and mock-inoculated samples detected by iTRAQ analysis for the specific protein associated with each pathway. P-values were calculated from the relationship between the differently expressed proteins identified by iTRAQ analysis and their associated KEGG pathways. There is an inverse correlation between the p-value and the number of differentially expressed genes

The total protein yield from the CGMMV-infected plants was much lower than that of the mock-inoculated control, being 3.8 μg/μL and 7.4 μg/μL, respectively. The results of the iTRAQ analysis revealed that the protein profile of the two treatments also differed greatly and identified 191 unique proteins only expressed in the inoculated plants. Statistical analysis combining the data from the GO [26] and KEGG [27] analyses was used to interpret the novel proteomic data in relation to genomic information contained in databases such as SwissPROT (http://www.expasy.org), GenBank (http://www.ncbi.nlm.nih.gov/genbank/), EMBL (http://www.embl.org) and DDBJ (http://www.ddbj.nig.ac.jp/). The results revealed that 38 proteins had significantly different expression levels. Of these, 35 were assigned function by the GO analysis (http://www.geneontology.org). According to the results, 22 of the proteins had a combined function being assigned to all three categories: molecular function (MF), biological process (BP), and cellular component (CC). Several other proteins were found to have dual function with 1, 7 and 2 proteins being assigned to BP/CC, BP/MF, CC/MF, respectively, while 3 proteins were found to be associated with a single function, namely MF (Table 1). The KEGG analysis (http://www.genome.jp/kegg/) identified 18 proteins that had roles in 13 separate pathways. Five of the pathways including ko00360, ko00940, ko00680, ko03013 and ko04146 were found to involve the up-regulation of proteins. In particular, pathways ko00360, ko00940, and ko00680, which corresponded to phenylalanine metabolism, phenylpropanoid biosynthesis and methane metabolism, respectively, were associated with a substantial up-regulation of five peroxidase proteins (Q39650, P19135, Q40559, Q39652 and Q39653). However, the majority of the pathways including ko04745, ko04530, ko04810, ko04510, ko04520, ko00630, ko00195 and ko00190 were associated with down-regulation (Fig. 2, Table 2).

**Table 1:** Proteins with significantly altered expression in cucumber plants inoculated with CGMMV relative to healthy mock-inoculated control plants

No	GPI	IP	AN	OT	FC
1	Q3EBC8.2	Isoform 2 of endoribonuclease Dicer homolog 2	-	-	−1.5
2	Q4VZK4	Cytochrome b6 - f complex subunit 4	GO:0051234, GO:0022900	BP	−1.4
			GO:0034357, GO:0031224 GO:0009534	CC	
			GO:0003824, GO:0009055	MF	
3	P62776	Histone H4	GO:0034728	BP	−1.2
			GO:0043231, GO:0000785	CC	
			GO:0003676	MF	
4	P08216	Malate synthase, glyoxysomal	GO:0009060, GO:0044262	BP	−1.1
			GO:0005777	CC	
			GO:0046912	MF	
5	P49296	Isocitrate lyase	GO:0009060, GO:0043436 GO:0044262	BP	−1.1
			GO:0005777	CC	
			GO:0016833	MF	
6	P48557	Histone H2B	GO:0034728	BP	−1
			GO:0000785, GO:0044444 GO:0043189	CC	
			GO:0003676	MF	
7	Q4VZI6	Cytochrome b6	GO:0051234, GO:0022900	BP	−0.8
			GO:0031224, GO:0009534	CC	
			GO:0003824, GO:0009055 GO:0005506	MF	
8	B8XX45	Actin isoform 2	GO:0043232	CC	−0.7
			GO:0032559	MF	
9	B8XX44	Actin isoform 1	GO:0043232	CC	−0.7
			GO:0032559	MF	
10	Q4VZH2	NAD (P) H-quinone oxidoreductase subunit J, chloroplastic	GO:0051234, GO:0008152	BP	−0.7
			GO:0009534	CC	
			GO:0048037, GO:0050136	MF	
11	Q4VZH1	NAD (P) H-quinone oxidoreductase subunit K, chloroplastic	GO:0051234, GO:0008152	BP	−0.7
			GO:0009534	CC	
			GO:0043169, GO:0051536 GO:0048037, GO:0050136	MF	
12	C1M2W0	Cinnamyl alcohol dehydrogenase	GO:0008152	BP	−0.7
			GO:0016616, GO:0046914	MF	
13	Q2QD76	Apocytochrome f	GO:0051234, GO:0006091	BP	−0.6
			GO:0009534, GO:0016021	CC	
			GO:0005506	MF	
14	Q84V66	Galactinol synthase	-	-	−0.6
15	Q8VWX5	Ribosome-like protein (^a^)	GO:0010467	BP	−0.6
			GO:0030529, GO:0009536	CC	
			GO:0003723, GO:0005198	MF	
16	A3F9M6	Phospholipase D	GO:0006650	BP	0.6
			GO:0043231, GO:0016020	CC	
			GO:0046872, GO:0004620	MF	
17	A4GWU2	Elongation factor 1-alpha (^a^)	GO:0006412, GO:0009207	BP	0.6
			GO:0044424	CC	
			GO:0008135, GO:0032561 GO:0017111	MF	
18	Q9SXL9	Csf-1 protein (^a^)	GO:0010467, GO:0022613	BP	0.6
			GO:0031090, GO:0009536 GO:0031981, GO:0015934	CC	
			GO:0005198	MF	
19	A1BQK8	Cysteine synthase (^a^)	GO:0006563, GO:0000097	BP	0.6
			GO:0044424	CC	
			GO:0016835, GO:0016765 GO:0048037	MF	
20	Q9FRW1	Disulfide isomerase (^a^)	GO:0018904, GO:0019725	BP	0.6
			GO:0044432	CC	
			GO:0016862, GO:0015036	MF	
21	Q39650	Truncated processed peroxidase (^a^)	GO:0016209, GO:0005506	MF	0.6
22	B3U2C1	Starch branching enzyme I	GO:0005982	BP	0.6
			GO:0009536	CC	
			GO:0016798, GO:0043167 GO:0016758	MF	
23	A1BQL2	Small ras-related protein (^a^)	GO:0023052, GO:0009207 GO:0015031	BP	0.6
			GO:0043231	CC	
			GO:0017111, GO:0032561 GO:0051169	MF	
24	B1PE19	Superoxide dismutase [Cu-Zn]-like	GO:0072593	BP	0.7
			GO:0044424	CC	
			GO:0003824, GO:0016209 GO:0043169	MF	
25	Q8RW69	Isocitrate dehydrogenase [NADP]	GO:0009060, GO:0044262 GO:0019752	BP	0.8
			GO:0009536	CC	
			GO:0046872, GO:0004448 GO:0000166	MF	
26	P19135	Peroxidase 2 (^a^)	GO:0042743, GO:0006950	BP	0.8
			GO:0016209, GO:0005506 GO:0003824	MF	
27	P83966	Monodehydroascorbate reductase, fruit isozyme (^a^)	GO:0008152	BP	0.8
			GO:0016655	MF	
28	B6V8E7	1a protein	GO:0001510, GO:0010467 GO:0033648, GO:0033644 GO:0044464	BP	0.8
			GO:0008173, GO:0017111 GO:0003676, GO:0032559	MF	
29	P29602	Cucumber peeling cupredoxin	GO:0051234, GO:0006091	BP	0.8
			GO:0046914	MF	
30	Q40559	Peroxidase	GO:0006950, GO:0008152	BP	0.9
			GO:0016209, GO:0005506 GO:0003824	MF	
31	Q2MK92	Acid alpha galactosidase 1	GO:0044238	BP	0.9
			GO:0015925, GO:0043167	MF	
32	Q8LK13	26 kDa phloem protein	-	-	0.9
33	O04379	Protein argonaute 1	GO:0009725, GO:0048364 GO:0042445, GO:0045087 GO:0009887, GO:0035194 GO:0048468, GO:0003006 GO:0009639	BP	0.9
			GO:0043231, GO:0044444	CC	
			GO:0003723, GO:0004519	MF	
34	Q39652	Peroxidase	GO:0016209, GO:0005506	MF	1
35	Q94G09	TASSELSEED2-like protein	GO:0009746, GO:0009756 GO:0006560, GO:0009688 GO:0006950	BP	1.1
			GO:0044444	CC	
			GO:0004022	MF	
36	Q39653	Peroxidase	GO:0016209, GO:0005506	MF	1.4
37	A1BQL5	Chitinase (^a^)	GO:0006026	BP	1.4
			GO:0044421	CC	
			GO:0004553, GO:0043167	MF	
38	Q5DJS5	Thaumatin-like protein	GO:0006950	BP	2.3
			GO:0043231	CC	

*GPI* Gene product information, *IP* Identified protein, *AN* Accession number, *OT* Ontology function, *BP* biological process, *CC* cellular component, *MF* molecular function, ^a^fragment (partial sequence). *FC* fold change in expression level (fold change >0.6). Negative values represent down-regulation

**Table 2:** Results of the KEGG analysis indicating the specific proteins impacting 13 metabolic pathways through their altered expression in CGMMV infected cucumber plants

No	PE	PN	PV	GPI
1	ko00360	Phenylalanine metabolism	0.0026	Q39650, P19135, Q40559, Q39652, Q39653
2	ko00940	Phenylpropanoid biosynthesis	0.0026	Q39650, P19135, Q40559, Q39652, Q39653
3	ko04745	Phototransduction - fly	0.0336	B8XX45, B8XX44
4	ko04530	Tight junction	0.0336	B8XX45, B8XX44
5	ko04810	Regulation of actin cytoskeleton	0.8910	B8XX45, B8XX44
6	ko04510	Focal adhesion	0.8910	B8XX45, B8XX44
7	ko04520	Adherens junction	0.8910	B8XX45, B8XX44
8	ko00680	Methane metabolism	0.0912	Q39650, P19135, Q40559, Q39652, Q39653
9	ko03013	RNA transport	0.2329	A4GWU2, A1BQL2
10	ko00630	Glyoxylate and dicarboxylate metabolism	0.3862	P08216, P49296
11	ko04146	Peroxisome	0.5266	B1PE19, Q8RW69
12	ko00195	Photosynthesis	0.7997	Q4VZK4, Q4VZI6, Q2QD76
13	ko00190	Oxidative phosphorylation	0.8436	Q4VZH2, Q4VZH1

*PE* pathway entry, *PN* pathway name, *PV p*-value, *GPI* gene product information

The thirteen pathways highlighted by the KEGG analysis were investigated further to determine their interrelationship during CGMMV infection. The KGML of each KEGG document was obtained from its corresponding KEGG webpage, and using the KEGG Parser software package [MathWorks, Massachusetts, U.S.] the information associated with the pathways and the data for individual proteins was extracted to an excel file [MergeKEGG, Kangyusheng, Shanghai, China]. The merged excel file was then imported into Cytoscape software [http://apps.cytoscape.org/apps/kgmlreader], which generated 3 separate network diagrams (Fig. 4). The nodes in the diagrams correspond to the individual metabolites associated with the pathways identified by the KEGG analysis, while the black lines illustrate the interrelationships between them. The results therefore show how the 13 KEGG pathways interact during CGMMV infection.

### Expression levels of 25 proteins identified by iTRAQ during CGMMV infection

**Fig. 3:**
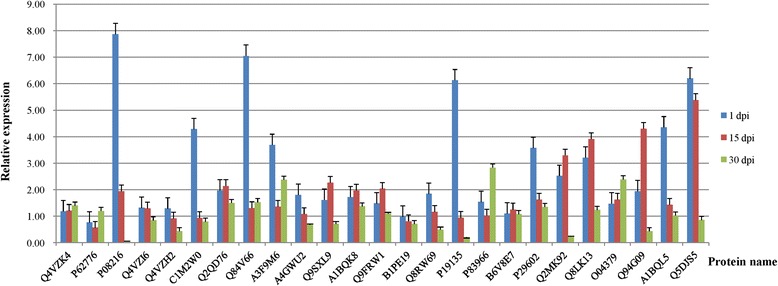
qPCR verification of 25 differentially expressed proteins identified by iTRAQ analysis showing their expression levels at 3 time points during CGMMV infection of cucumber plants

The expression levels of 25 proteins selected randomly from the iTRAQ analysis were assessed at three time points post inoculation (1, 10 and 30 dpi). The levels of several proteins including P08216, Q4VZI6, Q4VZH2, C1M2W0, A4GWU2, B1PE19, Q8RW69, P19135, P29602, A1BQL5 and Q5DJS5 were found to decline, while the levels of Q4VZK4 and O04379 were found to increase. Other proteins were found to exhibit more complex patterns of expression with Q2QD76, Q9SXL9, A1BQK8, Q9FRW1, B6V8E7, Q2MK92, Q8LK13 and Q94G09 being found to initially rise before falling, while P62776, Q84V66, A3F9M6 and P83966 were down-regulated before being up-regulated (Fig. 3).

## Discussion

The current study identified thirty-eight proteins with significantly altered expression in cucumber plants infected with CGMMV. Gene Ontology (GO) analysis (Table 1) classified the proteins into three groups based on their function, data that could be useful when investigating the function of proteins in vivo and screening their impact during CGMMV infection and ultimately in the development of disease resistant plants. The individual gene products were not allocated to single cellular processes, but were involved in several biological processes with multiple molecular functions [28, 29]. This effect is not uncommon; for example, the C-function activity associated with organ differentiation in *Medicago truncatula* is controlled by two eu*AG* genes, *MtAGa* and *MtAGb* [30], while conversely it has also been shown that single genes can control multiple phenotypes and different characteristics [28]. The down-regulation of cytochrome *b*6 (Q4VZK4, −1.4 fold change) in the current study, which affected the photosynthesis pathway (ko00195), is a further example of this phenomenon. Cytochrome *b*6 is an essential component of the cytochrome *b6-f* complex, which mediates electron transfer between photosystem I (PSI) and photosystem II (PSII), cyclic electron flow around PSI, as well as being an integral component of the chloroplast and thylakoid [31, 32].

Both in vitro and in vivo studies have demonstrated that the stresses caused by pathogens can alter the physiological characteristics of host plants [33]. The current study found that cucumber plants infected with CGMMV had significantly altered levels of phenylalanine metabolism (ko00360) and phenylpropanoid biosynthesis (ko00940). Phenylalanine was the most important component in the ko00360 and ko00940 pathways, and its metabolites such as flavonoids and lignin can play key roles in the resistance of plants to pathogen infection. Flavonoids are an important group of plant secondary metabolites that have been studied extensively using both physiological and molecular approaches [34]. It has been found that, in addition to acting as attractants for pollinators and seed dispersal agents as a result of their role in flower and fruit pigmentation, they also affect fertility and disease resistance [35]. For example, it has been shown that high levels of flavonoids in *Arabidopsis thaliana* can affect auxin transport and repress plant growth [36]. Similarly it has also been documented that the accumulation of high levels of lignin in cotton is linked to resistance to *Verticillium dahliae* [37]. Such studies indicate that the altered plant growth characteristics resulting from increased levels of flavonoids and lignin would be useful traits to consider when developing disease resistant varieties. Furthermore, the current study also found that the effects on phenylalanine metabolism (ko00360) and phenylpropanoid biosynthesis (ko00940) were linked to the well-characterized phenylalanine ammonia lyase (PAL) pathway (k10775), which is known to promote the metabolism of phenylalanine into monolignol, a process which is thought to play a key role in the cell wall lignification that enhances disease resistance by creating a barrier to the further invasion by pathogens [38]. Indeed, it has been shown that the over-expression of PAL in tobacco can enhance host resistance to infection by the fungus *Cercospora nicotianae* [39]. Plant peroxidases (PO) are also involved in lignin formation and represent another important factor in the resistance of plants to pathogen attack [40]. The current study found that both the ko00360 and ko00940 pathways were involved in the response of cucumbers to infection by CGMMV, with their expression being up-regulated 4.7 fold. Furthermore, the KEGG analysis revealed that the pathways associated with ko00360 and ko00680, which involved the up-regulation of 5 separate proteins, were the most significantly affected of all the pathways identified. All of the up-regulated proteins in these pathways were peroxidases, which implies that the over-expression of POs could be an important factor in the disease response of cucumber to CGMMV infection.

Several other proteins were also found to be up-regulated in the inoculated cucumber plants including the small ras-related protein (A1BQL2) and elongation factor 1-alpha (A4GWU2), which were both associated with the RNA transport pathway (ko03013), and it is interesting to note that both proteins have been associated with cucumber resistance in previous studies [41, 42]. Although not highlighted by the KEGG analysis, the up-regulation of phospholipase D (A3F9M6) was also of interest given that it has been associated with the defense signaling of *Arabidopsis* infected with powdery mildew (*Blumeria graminis* f. sp. *hordei*) [43], which indicates that it could also be an important component of the defense response of cucumbers.

However, the majority of the pathways (8 in total) were associated with the down-regulation of proteins. For example, it was found that the down-regulation of both isoforms of actin (B8XX44, B8XX45) impacted many pathways in the KEGG analysis (5 in total). Actin, which is evolutionarily highly conserved, is a key component of the cytoskeleton in all eukaryotes [44, 45] and plays an important role in protein-protein interactions [46, 47], as well as being involved in developmental processes, membrane trafficking and disease resistance [46, 48]. Meanwhile, the actin analogues in prokaryotes function as a cytoskeleton [49] that stabilizes the shape of the cell [50]. In the current study, both isoforms of (B8XX and BXX5) were found to be involved in the phototransduction pathway, which was significantly up-regulated. Given that previous studies have demonstrated that this process can affect the expression of chloroplastic genes [51], it is possible that the altered expression of actin could be associated with the chlorotic symptoms observed in CGMMV-infected leaves. It was also noted that both cytochrome b and cytochrome *b*6 were significantly down-regulated in the CGMMV infected plants. Cytochrome b (*cyt b*) is a membrane protein, which forms the core of the mitochondrial *bc*_1_ (complex III) in the respiratory chain, and is responsible for transmembrane electron transfer. The *cyt* b subunit possesses many inhibitor sites that can dramatically affect oxidoreductase activity as well as facilitate the action of quinone antagonists [52]. Consequently, *cyt b* has frequently attracted the attention of microbiologists [53], and it has been shown that in addition to its role in respiratory electron transport in the phytopathogenic bacteria *Pseudomonas cichorii* and *Pseudomonas aptata* [54], it is also associated with their pathogenicity [55]. In addition, previous studies have also shown that *cyt b* is associated with fungicide resistance, with two separate point mutations in the *cyt b* gene resulting in resistance to Qo inhibitors (QoIs) [56], which inhibit mitochondrial respiration by binding to the Qo site of complex III [57]. It is also interesting to note that the protein CmPP36, a member of the cytochrome b5 family, is required for the shuttle of Red clover necrotic mosaic virus (RCNMV) particles into the phloem translocation pathway [58], which has been shown to facilitate the systemic movement of CGMMV in infected cucumber plants [59]. This aspect of *cyt* b associated metabolism provides an intriguing opportunity for the development of control strategies to prevent CGMMV infection.

Meanwhile, the analogous protein *cyt b*6 is a component of the *cyt b*6f complex, which is associated with both PSI and PSII, and functions as a proton translocase in the cyclic electron transport of PSI. The *cyt b*6f complex is comprised of both nuclear and plastid encoded subunits, and it has been shown that nuclear mutants can result in defects in the *cyt b*6f complex that impair photosynthesis due to the uncoupling of electron transport [60]. Previous research has also shown that some viruses can affect the structure and function of the photosystems, reducing the rate of photosynthesis by as much as 60 %. Moreover, as an indispensable physiological process in all green plants, the altered photosystems also result in a reduction in the synthesis of cellular components in the affected plants [61–65], especially the synthesis of photosynthetic pigments, which has been associated with the symptom of virus infection in *Nicotiana tabacum* [66]. It is therefore possible that similar effects could explain the chlorosis and mosaic patterning in the leaves of CGMMV-infected cucumbers, while the alterations to actin and cytochrome b could account for the abnormal shape of the plants and fruits, which results in an indirect loss of yield [2].

Two of the most significantly down-regulated proteins were histones (P62776, P48557). Histone acetylation is important in the activation of genes at the posttranslational level, and a previous study [67] has shown that histone acetyltransferase (AtHAC1) can regulate flowering time in *Arabidopsis* via inhibition of Flowering Locus C (FLC). It is therefore possible that the down-regulation of the histones in cucumbers infected with CGMMV could be responsible for the delayed flowing that can indirectly affect production. It is also interesting that both malate synthase (P08216) and isocitrate lyase (*ICL*) (P49296) were down regulated in plants inoculated with CGMMV. Previous studies have shown that endogenous malate synthase is exploited by *Stagonospora nodorum* during its colonization of wheat [68], while isocitrate lyase is a key component of the glyoxylate cycle [69], and has been found to be the most important factor when *Leptosphaeria maculans* successfully colonizes canola [70]. It was also interesting to note that galactinol synthase (Q84V66) was down-regulated in the CGMMV inoculated plants, given that a recent study has shown that the overexpression of cucumber galactinol synthase (CsGolS) improved resistance to *Botrytis cinerea* in transgenic tobacco plants [71].

The relationships between host and pathogen have long been recognized to have a great influence on the evolution of the host and its biological diversity [72] as well as it resistance to pathogens [73]. The current study utilized qPCR to validate the expression of the differentially expressed proteins identified by the iTRAQ LC-MS/MS analysis at the transcriptional level, and assess the expression levels of the corresponding genes at 3 time points during CGMMV infection. All of the genes assessed exhibited altered patterns of expression compared to the mock-inoculated control, indicating that these genes were involved in the disease-response of cucumber plants to CGMMV infection. Of particular interest were the genes corresponding to O04379 (Protein argonaute 1) and Q4VZK4 (Cytochrome b6–f complex subunit 4), which exhibited increasing levels of expression as the CGMMV infection progressed. Previous research [74] has shown that protein argonaute 1 (AGO1), which is an essential component in a multitude of developmental processes including those of leaves, floral organs and the formation of axillary meristems, also has a role in RNA-mediated post-transcriptional gene silencing (PTGS) and antiviral RNA silencing, which indicates that the elevated levels of O04379 could be associated with CGMMV-resistance in cucumber. In contrast, the increased expression of Q4VZK4, which is a component of the cytochrome b6-f complex (cyt-b6f) that mediates electron transfer between photosystem II(PSII) and photosystem I(PSI) [75], is more likely to be associated with the disease-response of the cucumber host and the symptoms of CGMMV infection. Cyt-b6f is located in the thylakoid membranes of plant chloroplasts [76], and previous research has shown that the coat protein of tobacco mosaic virus (TMV-CP), which like CGMMV belongs to the *Tobamovirus* genus, can become embedded inside or otherwise complex with the thylakoid membranes impairing their function [77].

**Fig. 4:**
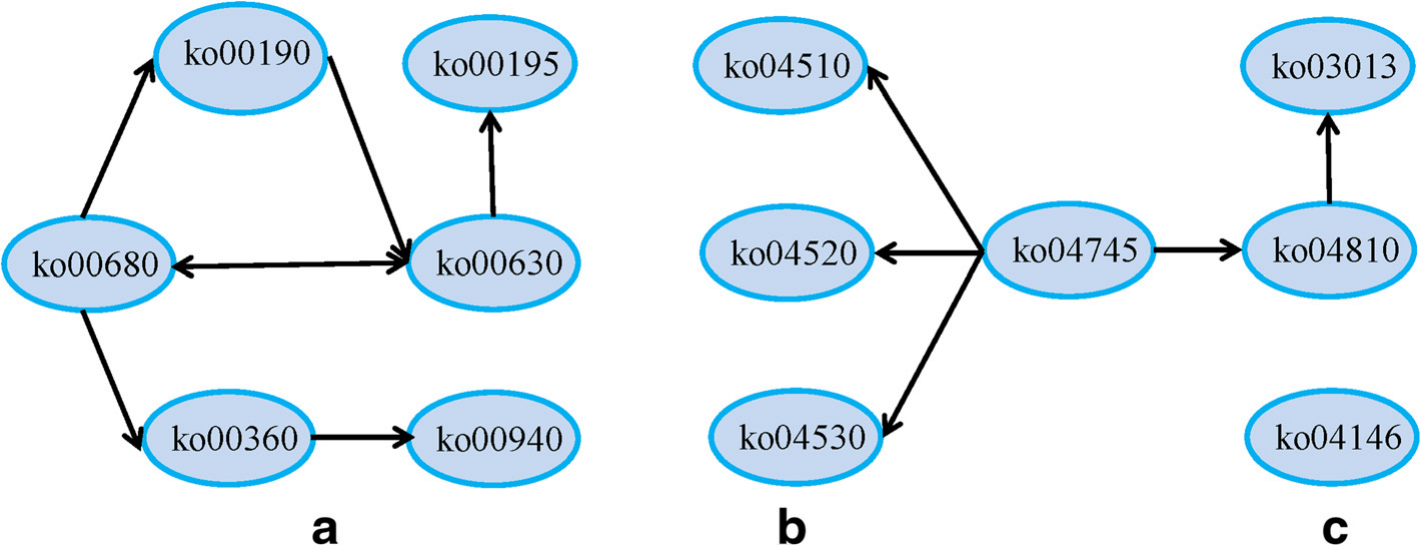
Three merged network diagrams (**a**, **b**, **c**) illustrating the intricate relationships between the 13 KEGG pathways associated with CGMMV-infected cucumber leaves. Each node in the figure represents an individual metabolite, while the lines illustrate the interactions and shared components of the separate KEGG pathways in CGMMV-infected cucumbers. **a** the diafram includes ko00940, ko00360, ko00680, ko00190, ko00630 and ko00195 pathways; **b** the diagram includes ko04745, ko04520, ko04530, ko04510, ko04810, ko03013 pathways; **c** only one pathway named of ko04146

While GO annotation can establish the basic function of particular proteins, KEGG analysis can be used as an indication of the proteins role within a network of interacting molecules in the cell. This can be a very useful tool to elucidate the role of genes of interest. The merged network diagrams generated in the current study highlight the relationships between the annotated proteins and the metabolic pathways that they impact. The current study found 38 proteins that were differentially expressed in CGMMV-infected cucumber and highlighted the role of 18 differentially expressed proteins in the context of 13 separate metabolic pathways. Based on the results of the GO annotation and the KEGG analysis these 13 KEGG pathways were selected for the merged analysis performed using Cytoscape software, which provided an insight into how the different KEGG pathways interacted (Fig. 4). The results highlighted several pathways that were significantly affected by CGMMV infection including phenylalanine metabolism (ko00360), phenylpropanoid biosynthesis (ko00940), and methane metabolism (ko00680), which were all characterized by a 4.7 fold up-regulation, as well as glyoxylate and dicarboxylate metabolism (ko00630), which were 2.2 fold down-regulated. The regulatory mechanisms induced during the response of plants to disease infection are complex and operate at physiological, cellular and transcriptome levels [78]. The results of the current study showed that CGMMV infection affected not only regulatory mechanisms specific to disease response but also had a crossover effect with other regulatory mechanisms. For example, L-phenylalanine and L-tyrosine both mediated the interactions between pathways ko00360 and ko00940, while pyruvate was associated with the function of the ko00360 and ko00680 pathways (Fig. 4a). Statistical analysis revealed that pathways ko00360 and ko00940 were the most significantly affected producing a *p*-value of 0.0026. Furthermore, the network diagrams illustrated in Fig. 4 shown that pathway ko04745 can affect other pathways including ko04510, ko04520 and ko04530 via protein-protein interactions (Fig. 4b). The network of relationships identified by such studies provides valuable data regarding plant pathogen interactions and can highlight potential areas for further study. Furthermore, they also suggest that research should not be focused on single regulatory factors but rather the interaction of a range of regulatory mechanisms via various processes including gene expression as well as enzyme-enzyme, protein-protein, and protein-metabolite interactions. It is likely that such an approach would greatly increase our understanding of the regulatory network operating during the CGMMV infection of cucumber plants, and could provide useful clues that could facilitate the long term control of CGMMV by the development of resistant varieties.

## Methods

### Sample preparation and protein extraction

Cucumber (*Cucumis sativus*) seedlings (cv. Zhongnong 16) obtained from the Institute of Vegetables and Flowers at the Chinese Academy of Agricultural Sciences, (Beijing, China) were grown in sandy soil fertilized with chicken manure. Plants were inoculated with CGMMV obtained from the Chinese Academy of Inspection and Quarantine (CAIQ) at the three true-leaf stage. The plants were grown in a greenhouse at the China Agricultural University under natural light conditions with the temperature maintained between 21 °C and 32 °C, and watered when necessary. The plants were kept in net cages (Zhiguang wire mesh products, Hebei, China) to prevent insect feeding and cross infection by other pathogens. Mock-inoculated control plants inoculated with phosphate buffer (0.05 M, pH7.0) were prepared and maintained under identical conditions. The plants were assessed after 45 days when the inoculated plants displayed typical symptoms of CGMMV infection including yellowing, as well as mottled and mosaic patterning of the leaves. The presence of CGMMV in leaves exhibiting symptoms of disease was confirmed by SEM and RT-PCR as described in a previous study [2]. The proteins from the leaves of the control and CGMMV-positive plants were extracted according to the protocol of Fan [79] and quantified using the RC DC™ Protein Assay Kit [Bio-Rad, CA, U.S.] according to the protocol of the manufacturer.

### Protein processing and iTRAQ labeling

The extracted protein samples were transferred to fresh 1.5 mL centrifuge tubes and suspended in 250 μL sample buffer (8 M UREA, 4 % CHAPS, 30 mM HEPES, 1 mM PMSF, 2 mM EDTA, 10 mM DTT). The samples were dissolved completely using a KQ-250DE ultrasonic sonicator (Kun Shan, China) set to pulse on 2 s, pulse off 3 s, power 180 W. After centrifugation at 12,000 rpm for 25 min, the supernatant was collected and transferred to fresh tubes before 20 μL of 55 mM iodoacetamide (IAM) was added to each tube. After dark-incubation in a water bath at 56 °C for 1 h, four times the sample volume of cold acetone was added to each tube, and the samples precipitated at −20 °C for 3 h. The samples were then centrifuged at 13,000 rpm for 15 min at 4 °C and the supernatant discarded. The protein pellet produced was dissolved in 300 μL dissolution buffer (0.5 M TEAB, 0.1 % SDS) and the quantity of the protein determined by the Bradford method. The prepared soluble protein samples were stored at −80 °C until required.

Protein digests were prepared by adding 3 μg trypsin Gold [Promega, Madison, U.S.] to 100 μg of extracted proteins using a 0.5 M TEAB, 0.1 % SDS solution to balance the sample volume, and incubated at 37 °C for 24 h. The digested samples were re-dissolved in 0.5 M TEAB and labeled with isobaric tags using the iTRAQ Reagents Multiplex Kit [Applied Biosystems Inc., CA, U.S.] according to the protocol of the manufacturer. The samples were labeled using 119 and 121 tags for the CGMMV-positive and mock-inoculated control samples, respectively.

### Separation and identification of peptide fragments

The two samples, which had been labeled separately with either the 119 or 121 tag, were mixed, re-suspended and diluted 10-fold in 25 % acetonitrile (ACN), 10 mM potassium dihydrogen phosphate (KH_2_PO_4_), adjusted to pH 3.0 using phosphoric acid, and purified using a C18 reverse-phase LC column (Shimadzu corporation, Japan) to remove salts and the by-products of the iTRAQ reaction. The samples were washed and eluted according to the protocol of the manufacturer, and the eluates dried down and fractionated using a Luna®5 μm, 100 A, 250 × 4.60 mm strong cation exchange (SCX) column [Phenomenex Inc., CA, U.S.]. The peptides were eluted at a flow rate of 1.00 mL min^−1^, with a linear gradient of 0–100 % solvent B (10 mM KH_2_PO_4_, 2 M KCl, 25 % ACN, pH3.0) over 86.1 min, and the peptide fraction collected for use in the subsequent analysis.

The peptide fractions were centrifuged and dried at low temperature before being re-suspended in 0.1 % formic acid, and ionized by matrix-assisted laser desorption/ionization (MALDI). The resulting ions were then detected using the Agilent 6550 iFunnel Q-TOF LC/MS mass spectrometry system (Agilent Technologies Inc., U.S.). The data collected was used to search online databases using the MASCOT software package (Matrix Science, Inc., Boston, U.S.) to identify the component proteins. The data and search results were then imported into Scaffold software (http://www.proteomesoftware.com/) [Proteome Software, Inc., Portland, U.S.] for protein quantification, and the results verified by analysis of variance (ANOVA) and the student’s *T*-test using the SPSS 19.0 software package [IBM, CA, U.S.]. The variation generated between the CGMMV-infected and mock-inoculated samples was used to estimate the level of differential protein expression. The analysis was made using two biological replicates, with an absolute value for the fold change of greater than 0.6 indicating a differentially expressed protein.

### qPCR analysis of 25 differentially expressed proteins identified by iTRAQ analysis

The leaves (100 mg) of CGMMV-infected and mock-inoculated cucumber plants were collected at three time points post inoculation: 1, 15 and 30 dpi. The samples were ground to a fine powder in liquid nitrogen using a pestle and mortar and the total RNA extracted using TRIzol Reagent (Invitrogen Life Technologies) before the cDNA was prepared (cDNA synthesis kit, Invitrogen, U.S.) using 3 μg RNA according to the protocol of the manufacturer. The primers listed in Table 3 were used to compare the expression levels of 25 proteins selected at random from the 38 proteins identified by the iTRAQ analysis using tubulin as the internal standard. The qPCR itself was conducted in 25 μl reaction mixtures containing 1.0 μl cDNA, 0.5 μl each of the forward and reverse primers (10 μM), 12.5 μl 2 × TaqMix, and 1.25 μl SYBR Green (BIO-RAD, U.S.), and processed using a real-time PCR thermocycler (BIO-RAD, U.S.) with the following program: denaturation at 95 °C for 5 mins followed by 40 cycles of denaturation at 95 °C for 15 s, annealing for 20 s, and extension at 72 °C for 20 s. The annealing temperature used was specific to individual primer sets depending on their GC content (Table 3). A total of three biological replicates were performed (SD <0.2).

**Table 3:** Primers used to confirm in vivo expression of 25 proteins identified by iTRAQ analysis of cucumber plants infected with CGMMV

Protein name	Primer sequence (5’-3’)	Annealing temperature (°C)
Q4VZK4	TGAGACGACCCAGAAAGCACT	59.6
	GCACAAATCAAACGAACCAAC	58.1
P62776	AAGGAGGAGCAAAGAGACACA	56.5
	GGCATAAACAACATCCATAGCAGT	60.4
P08216	ATGGAGATGGGCTTGGTGTGA	62.3
	GCTGTGCATTGCCTTGTGAAC	61.4
Q4VZI6	AGAAGATTCAAGAGGCCCCTA	57.5
	GGCTCAAACAGAAACACCCAA	60.0
Q4VZH2	CTTCCATTCCATTCCTTTCCA	59.1
	CATTACTCTCCTTTTTCTTGCG	56.8
C1M2W0	CAAAAGTGGGCTAAGAGGAGG	58.5
	TGTCGATGATGTAGTCAAGAGAGT	55.9
Q2QD76	ACCTTTCCTATTCTTTCCCCG	59.4
	TACTTACTATCCCTGCTGCTGT	54.2
Q84V66	AGGTACACGGGAAAAGAAGAG	55.6
	TCGGACAGAACAGAGATCAAA	55.4
A3F9M6	ACCCTTTCACTCCCTTTTCAG	57.7
	CATCCTTCCCACCTTGTTTCT	58.2
A4GWU2	ACTGGTGGTTTTGAGGCTGGT	58.0
	CTTGGAGTATTTGGGTGTGGT	56.0
Q9SXL9	GGGATGTTCAGAAGAAATGGG	58.5
	GAAAGTCGAAAGAGCAAAAGGA	58.5
A1BQK8	GGATGAAGGTGTAGTTGATGAA	54.3
	ATGGAAAAGGGCAGAGGATAG	57.8
Q9FRW1	AAGTGCCTGTCCTTCTTGTGC	59.3
	TGTTACTCTCGGGAATGGGTT	58.7
B1PE19	TCTTGGAAGTAGTGAGGGTGTT	55.7
	ATCTGGCAGTCAGTAATGGTG	54.9
Q8RW69	ATGACTTCGGTGTTGGTTTGT	56.9
	CTATGCTGTTGGTGCTTGTTT	55.7
P19135	TTTGGTACTGTGTTTGATGATGGC	61.7
	TCGTGAAAATGGAGACGGATG	60.7
P83966	CTTGAGATGAACAAAGGGGGG	61.0
	CACGATAGATCGAAAACGCGC	63.3
B6V8E7	CAGTTCATTCCACACCCTATC	54.3
	GTGCTCATTTTCTTGACCCTT	56.2
P29602	AGACTCGACGAACTCGGCATG	62.5
	CGGAGAAAGTGAGATAAAGGG	56.1
Q2MK92	GGCGATAAATTAGGAAACAGC	56.6
	AATCACAACCAAGAAGAAGGG	56.0
Q8LK13	TCCTACTTCCAAAATCCCTCA	56.6
	TATTGCTTCAACTTTCCACTTATAT	55.3
O04379	TATTTTACAGGGATGGGGTTAG	56.0
	ACTGGAGGTTGGTAGTTTGGT	55.4
Q94G09	GTCAGTCATCTTCTGTCTTCGTT	55.4
	TCATTACTCGTGCTGCGTGTT	59.2
A1BQL5	TCGTCAACATAGCATTTCTCTCATC	60.6
	AACGCCATCCAAAACCGCAGC	68.9
Q5DJS5	CATTCTGCCTTTGTGCTTTTTC	59.6
	ATTGATCGTCACGGTCTCGCC	64.4
Tubulin	CAAGGAAGATGCTGCCAATAA	58.0
	CCAAAAGGAGGGAGCCGAGAC	60.0

## Conclusion

In conlusion, total of 38 proteins were identified for the first time, and the expression level of 23 proteins were up-regulated and 15 proteins down-regulated in cucumber in response to CGMMV infection. Among of them, 18 proteins were involved in 13 individual metabolic pathways. The analysis results that 13 pathways also have crossing relation between of each other. These results provide the basic of investing the differences in protein expression and bring new insights into the relation of CGMMV and cucumber.

## Abbreviations

BP: biological process
CC: cellular component
CGMMV: cucumber green mottle mosaic virus
GO: gene ontology
iTRAQ: isobaric tags for relative and absolute quantitation
KEGG: kyoto encyclopedia of genes and genomes
LC-MS/MS: liquid chromatography-tandem mass spectrometric
MF: molecular function
